# Continuities and Discontinuities in the Cognitive Mechanisms Associated With Clinical and Nonclinical Auditory Verbal Hallucinations

**DOI:** 10.1177/21677026211059802

**Published:** 2022-01-17

**Authors:** Peter Moseley, Ben Alderson-Day, Stephanie Common, Guy Dodgson, Rebecca Lee, Kaja Mitrenga, Jamie Moffatt, Charles Fernyhough

**Affiliations:** 1 Department of Psychology, Northumbria University; 2 Department of Psychology, Durham University; 3 Tees, Esk, & Wear Valley National Health Service (NHS) Foundation Trust, West Park Hospital, Darlington, England; 4 Cumbria, Northumberland, Tyne, & Wear NHS Foundation Trust, St. Nicholas Hospital, Newcastle upon Tyne, England; 5 School of Psychology, University of Sussex

**Keywords:** hallucinations, psychosis, psychotic-like experiences, cognition, auditory perception, open data

## Abstract

Auditory verbal hallucinations (AVHs) are typically associated with schizophrenia but also occur in individuals without any need for care (nonclinical voice hearers [NCVHs]). Cognitive models of AVHs posit potential biases in source monitoring, top-down processes, or a failure to inhibit intrusive memories. However, research across clinical/nonclinical groups is limited, and the extent to which there may be continuity in cognitive mechanism across groups, as predicted by the psychosis-continuum hypothesis, is unclear. We report two studies in which voice hearers with psychosis (*n* = 31) and NCVH participants reporting regular spiritual voices (*n* = 26) completed a battery of cognitive tasks. Compared with non-voice-hearing groups (*n*s = 33 and 28), voice hearers with psychosis showed atypical performance on signal detection, dichotic listening, and memory-inhibition tasks but intact performance on the source-monitoring task. NCVH participants, however, showed only atypical signal detection, which suggests differences between clinical and nonclinical voice hearers potentially related to attentional control and inhibition. These findings suggest that at the level of cognition, continuum models of hallucinations may need to take into account continuity but also discontinuity between clinical and nonclinical groups.

Auditory verbal hallucinations (AVHs, or “voices”) are typically associated with schizophrenia or other psychotic disorders (Bauer et al., 2011) but are not specific to any diagnosis (Toh et al., 2015; Waters & Fernyhough, 2017). Evidence suggests that compared with nonhallucinating patients, individuals with schizophrenia and AVHs show biases or impairments in several cognitive domains, including reality monitoring (RM; memory for the self/nonself source of information; Brookwell et al., 2013; Woodward et al., 2007), auditory signal detection (SD; argued to reflect the influence of top-down processes; Bristow et al., 2014; Vercammen et al., 2008), attentional control (Hugdahl et al., 2013), and memory inhibition (Waters et al., 2003). Prominent cognitive models of AVHs accordingly suggest that they result from an externalizing bias in RM and/or overweighting of top-down processes (Moseley et al., 2013; Waters et al., 2012), which lead to external misattribution of self-generated mental events (e.g., inner speech). Atypical attention and inhibitory processes have been proposed to underlie the uncontrollable and intrusive elements of AVHs (Waters et al., 2003). Together, these mechanisms have been proposed as part of an influential multicomponent model of AVHs (Waters et al., 2012).

One approach to studying hallucinations outside of psychopathology has been to administer self-report questionnaires to assess variability in “hallucination proneness” in general-population samples. This approach avoids confounds of antipsychotic medication usage and comorbid symptoms of psychosis. Such studies have shown mixed results regarding associations between cognition and hallucination proneness in the general population. A number of studies have provided evidence that biased performance on auditory SD (Bentall & Slade, 1985; Brookwell et al., 2013; Moseley et al., 2021) or on other similar tasks (de Boer et al., 2019) is associated with hallucination proneness in the general population, whereas evidence regarding other cognitive domains is more mixed (Alderson-Day et al., 2019; Badcock & Hugdahl, 2012; Laroi et al., 2004; Moseley et al., 2021; Woodward et al., 2007). An alternative approach used in recent research has been to focus on individuals reporting AVHs of comparable frequency and recurrence to people with psychosis but who do not meet criteria for any psychiatric disorder (Peters et al., 2016; Powers, Kelley, & Corlett, 2017; Sommer et al., 2010). Such nonclinical voice hearers (NCVHs) tend to report fewer negative symptoms and less threatening appraisals (Peters et al., 2017), and their experiences tend to be less distressing and more controllable than in psychosis (Daalman, Boks, et al., 2011; Powers, Kelley, & Corlett, 2017).

Research into the cognitive mechanisms underlying AVHs in NCVHs is more limited, however. Daalman, van Zandvoort, et al. (2011) administered a neuropsychological battery to a group of NCVHs and reported lower scores (compared with non-voice-hearing control participants) on executive function, working memory, abstract reasoning, and a verbal intelligence assessment but not on long-term verbal memory, spatial reasoning, or processing speed. Neuroimaging has also indicated that NCVHs do not show atypical language lateralization in a verbal-fluency task (Diederen et al., 2010), which is commonly observed in schizophrenia (Sommer et al., 2001). Others have provided evidence for overweighted top-down processes in NCVHs (Alderson-Day et al., 2017; Powers, Mathys, & Corlett, 2017), although there is mixed evidence regarding structural differences in the paracingulate sulcus, a brain region involved in RM, across clinical and nonclinical groups (Garrison et al., 2019; Powers et al., 2020). However, although studies have investigated these aspects of cognition in relation to general-population hallucination proneness, to our knowledge, no study has reported on RM, intentional inhibition, or dichotic listening (DL; assessing both language lateralization and attentional control) in NCVHs or used the most common task linked to top-down processing in hallucinations research (auditory SD) in an NCVH group. Research in this area is crucial to untangle when atypical patterns of performance are specific to AVHs broadly, as opposed to psychotic AVHs specifically, or psychopathology more broadly.

We report on data from two studies regarding cognition in AVHs in psychosis and in NCVHs covering the core mechanisms reviewed above. As part of a larger ongoing study (Alderson-Day et al., 2021), we recruited individuals in early intervention in psychosis services (hereafter referred to as the *patient* group) reporting distressing AVHs (Study 1). Following prior research from other teams (Baumeister et al., 2017; Peters et al., 2016; Powers, Kelley, & Corlett, 2017), we also recruited NCVHs who reported hearing spiritual voices (often referred to as “clairaudient” or “psychic”); participating individuals reported regular voices but did not meet criteria for a psychiatric diagnosis (e.g., they were not distressed; Study 2). The patient and NCVH groups were compared with matched control participants on four cognitive tasks: source memory (which assesses RM; Woodward et al., 2007), auditory SD (Bristow et al., 2014), consonant–vowel DL (Hugdahl et al., 2013), and intentional inhibition (Waters et al., 2003). These tasks were chosen as some of the most frequently used in hallucinations research with psychosis patients and as key components of previous cognitive models (Waters et al., 2012) yet have not previously been used with NCVH samples (not including general-population proneness studies). NCVH participants also completed assessments of hallucinations, delusions, anxiety, and depression to assess other aspects of psychopathology compared with the general population. We expected that consistent with previous research, participants in the patient group would show atypical performance on all four tasks. If nonclinical experiences result from the same underlying mechanisms as psychosis, NCVHs would also show atypical performance on all four tasks. If underlying mechanisms are not continuous across the nonclinical/clinical divide, we would expect a different performance profile in NCVHs compared with patients. We set out to provide further data regarding links between cognition and hallucinations in both psychosis and NCVHs.

## Method

### Participants

A power analysis suggested at least 26 participants per group for comparisons using independent samples *t* tests, assuming a large effect size given previous meta-analytic evidence (Brookwell et al., 2013; α = .05, *d* = 0.70, power = 0.8), although recruitment proceeded flexibly based mainly on success recruiting into the voice-hearing groups. For Study 1, the psychosis-voice-hearer group (the patient group) consisted of service users recruited from early intervention in psychosis services in northern England (*N* = 31; mean age *M* = 28.55 years, *SD* = 10.22; *n* female = 14). All were of White British ethnicity (reflecting regional norms, given low racial diversity in this area of the United Kingdom). Service users were invited to take part if they were 16 to 65 years old, reported hearing voices at least once a week over the previous month, were fluent English speakers, had normal/corrected-to-normal vision, and were within their first 9 months of using early intervention services (because of participation in another study of which this was an inclusion criteria). Exclusion criteria were the presence of neurological diagnoses, hearing impairments, or suspected duration of untreated psychosis of more than 5 years. Information regarding diagnosis and medication usage is provided in Tables S1 and S2 in the Supplemental Material available online. The control group (*N* = 33; mean age = 27.91 years, *SD* = 10.41; *n* female = 19) was recruited using community advertisement, social media, and word of mouth.

For Study 2, the NCVH group was recruited from spiritualist communities across the United Kingdom (*N* = 26; mean age = 58.72 years, *SD* = 11.72; *n* female = 18) through newsletters, online advertisements, and visits by researchers to spiritualist churches. Individuals were invited to participate if they reported hearing voices at least once a month that did not solely occur within a spiritualist church. This latter criterion was used to ensure participants were not solely reporting experiences associated with meditation or trance. Participants were screened via telephone. Exclusion criteria were the same as for Study 1, with the addition of exclusion because of psychiatric diagnosis or severe distress. Specifically, participants were asked (a) if they ever found voices distressing, (b) if they had ever received a psychiatric or neurological diagnosis, and (c) if they had ever been in contact with health services regarding their voices. An affirmative answer to any of these questions led to exclusion from the study. The non-voice-hearing control group (*N* = 28; mean age *M* = 58.68 years, *SD* = 11.60; *n* female = 17) were recruited as in Study 1. Further demographic information is presented in Table 1.

**Table 1.** Demographic Information and Assessments of Intelligence in Studies 1 and 2

Characteristic	Study 1			Study 2		
	Patients	Control participants	Difference [95% CI]	NCVHs	Non-voice-hearer control participants	Difference [95% CI]
*N*	31	33	—	26	28	—
Age (years)	28.55 (10.22)	27.91 (10.42)	0.64 [−4.52, 5.80]	58.72 (11.72)	58.69 (11.60)	0.03 [−6.59, 6.54]
Gender (*n* female)^a^	14	19	0.50 [−0.49, 1.49]	18	17	−0.31 [−1.50, 0.88]
Education (years)	12.36 (1.85)	12.88 (1.45)	−0.29 [0.35, 1.39]	14.92 (3.12)	15.42 (2.24)	0.50 [−1.07, 2.07]
Matrix Reasoning^b^	15.37 (4.17)	18.46 (2.79)	3.09 **[1.32, 4.86]**	16.58 (4.76)	20.26 (2.85)	3.68 **[1.39, 5.98]**
NART^c^	23.32 (11.07)	30.21 (6.26)	6.89 **[2.43, 11.35]**	33.32 (10.10)	39.13 (6.92)	5.81 **[0.74, 10.88]**

Note: Values are means (with standard deviations in parentheses) unless otherwise specified. The 95% confidence intervals (CIs) represent the interval around the difference between the two groups’ means. Boldface type indicates that 95% CIs do not cross 0. NCVHs = nonclinical voice hearers.

a For this row, the values are number of female participants, and the 95% CIs are for the log odds ratio.

b Matrix Reasoning is from the Wechsler Abbreviated Intelligence Scale (Wechsler, 1999, 2008); scale range = 0–30.

c NART = National Adult Reading Test (Nelson, 1982); scale range = 0–50.

### Assessment of hallucinations and delusions

Psychotic Symptom Rating Scale. The Psychotic Symptom Rating Scale (PSYRATS; Haddock et al., 1999) is an interviewer-administered symptom-rating scale that provides scores for attributes relating to auditory hallucinations (11 items) and delusions (six items), including frequency, duration, location, loudness, and distress (for hallucinations) and preoccupation, conviction, distress, and disruption (for delusions). Sum scores on the auditory hallucinations scale can be calculated for cognitive (scored between 0 and 12), emotional (scored between 0 and 16), and physical (scored between 0 and 12) attributes. Both hallucinations and delusions subscales were used in the patient group (Study 1), although only the hallucinations subscale was used for the NCVH group (Study 2) because it was judged inappropriate to pathologize spiritual beliefs and also complex to unpick what could be classed as delusional ideation in the nonclinical group.

Launay-Slade Hallucination Scale. The Launay-Slade Hallucination Scale (LSHS; Bentall & Slade, 1985; McCarthy-Jones & Fernyhough, 2011) is a nine-item self-report scale assessing hallucinatory experiences and has subscales for auditory experiences (five items; e.g., “I have been troubled by hearing voices in my head”) and visual experiences (four items; e.g., “I see shadows and shapes when nothing is there”). Participants are asked to respond on a 4-point Likert scale (1 = *never*, 4 = *almost always*); scores range from 5 to 25 for the auditory subscale and 4 to 16 for the visual subscale. Unlike the PSYRATS, the LSHS is suitable for use across both general-population and clinical samples. Internal reliability in previous studies has been satisfactory (McCarthy-Jones & Fernyhough, 2011).

Peters Delusion Inventory. The Peters Delusion Inventory (PDI-21; Peters et al., 2004) is a 21-item self-report scale assessing delusional ideation (e.g., “Do you ever feel as if you are being persecuted in some way?”) with yes/no as response options (score range = 0–21). If participants respond yes, they are prompted to provide ratings for distress, preoccupation, and conviction on a 5-point Likert scale (score range for each subscale = 0–105). The scale has previously been used in both clinical and general-population samples and shown high internal reliability (Peters et al., 2004). This scale was used in both Studies 1 and 2—unlike the PSYRATS, the PDI-21 requires participants to answer a series of specific questions regarding specific topics of delusional ideation and so does not require rating, for example, beliefs regarding spiritualism.

Hospital Anxiety and Depression Scale. The Hospital Anxiety and Depression Scale (HADS; Zigmond & Snaith, 1983; Study 2 only) is a commonly used 14-item self-report scale assessing anxiety (seven items; e.g., “In the past month, I have felt tense and wound up”) and depression (seven items; e.g., “In the past month, I have looked forward to things with enjoyment”). Each item is scored on a 4-point scale (0–3); scores range from 0 to 21 for each subscale. Internal reliability has previously been shown to be satisfactory (Zigmond & Snaith, 1983). To reduce load for the patients, the HADS was administered only in Study 2 (although patients would have almost certainly scored higher than control participants on this measure, it was not of primary interest in this study).

### Cognitive tasks

National Adult Reading Test. The National Adult Reading Test (NART; Nelson, 1982) was used for descriptive purposes as a brief assessment of premorbid intelligence. Participants were required to read aloud from a list of 50 words in which correct pronunciation differs from the spelling; scores are given for correct pronunciation. Possible scores range from 0 to 50.

WASI Matrix Reasoning. The WASI Matrix Reasoning (MR; Wechsler, 1999), taken from the Wechsler Adult Intelligence Scale (Wechsler, 2008), was used as a brief assessment of nonverbal reasoning. Participants were required to complete a series of up to 30 pattern completion trials; possible scores range from 0 to 30.

The auditory SD required participants to detect a speech clip embedded in pink noise, presented through over-ear headphones (Sennheiser HD201). The protocol was similar to that used in a number of previous studies (Barkus et al., 2007; Moseley et al., 2014, 2021). Participants were presented with 80 3.5-s bursts of noise; a 1.5-s speech clip was presented at one of four intensities in 48 trials (speech-present trials), and no speech clip was embedded in 32 trials (speech-absent trials). The intensity (volume) of the speech clips in the speech-present trials was determined in pilot testing separately for each study and was set at detection rates of 25%, 50%, 75%, and 95% in pilot testing. Note that given the expected age gap between participants in the two studies, this task was calibrated separately for each study (i.e., the signal-to-noise ratio was higher for Study 2, for older participants), and so performance on this task is not directly comparable between studies. Specifically, given pilot testing and previous research by other groups (e.g., Powers, Kelley, & Corlett, 2017), we expected the NCVH group to be older than the patient group (recruited from early intervention services). Signal-to-noise ratios were therefore based on pilot testing of two groups: 10 participants ages 18 to 40 (for Study 1) and 10 participants ages 40 to 75 (for Study 2). It was not appropriate to set signal-to-noise ratios on a by-participant basis (i.e., run separate calibrations for each participant) because this would eliminate individual differences in, for example, sensitivity—a key variable we aimed to investigate. In each study, each voice-hearing group (i.e., the patient group and the NCVH group) was therefore presented with exactly the same stimuli as their respective control groups so that differences in SD parameters between groups could be explored. In the main task, after each trial, participants were asked whether they believed speech was present or not and responded yes/no with a button press. The primary outcome variable was false alarm rate (the proportion of speech-absent trials on which the participant responded yes); further analysis was also conducted on SD parameters for sensitivity (*d*′), calculated as the standardized hit-rate minus the standardized false alarm rate, and bias (β), calculated as

$$\beta =e{\left\{\frac{{\left[Z(F)\right]}^{2}-[Z(H)]}{2}\right\}}^{2}$$.

For both studies, performance was compared using independent samples *t* tests or Mann-Whitney *U* tests for nonnormally distributed data.

The source memory task (assessing RM) required participants to recall whether previously presented words had been presented as spoken stimuli through headphones (heard items) or whether they had themselves spoken the word (said items). One hundred twenty words were split into six lists of 20; stimuli were selected from previous studies that had employed a source-memory task (Moseley et al., 2018). In the encoding stage of the task, participants were presented with two of the lists (40 items), assigned as heard and said items. Participants were cued to either listen to or speak aloud each word (3.5 s per item, presented in a random order). In the recall stages, participants were presented with the same words plus words from a third list (20 new items). They were asked whether they believed each item was originally heard or said or was a new item, and they responded with a button press. The primary outcome variable for this task was the number of said items in the recall stage that were incorrectly recalled as heard (say-to-hear errors). Further analysis was conducted with the proportion of items that were correctly recalled as old for which the source was also correctly recalled (RM accuracy) and for the proportion of items that were correctly recalled as old or new (old-new recognition accuracy). As previously recommended (Woodward et al., 2007; Woodward & Menon, 2011), we analyzed group differences in both studies using analysis of covariance (ANCOVA) with say-to-hear errors as dependent variable, group as independent variable, and new-to-hear errors as a covariate (to correct for errors due to guessing).

The consonant–vowel DL task presented participants with conflicting single-syllable verbal stimuli to each ear simultaneously; stimuli were taken from previous research with schizophrenia patients (Hugdahl et al., 2012). Across three conditions, participants were required to (a) select the syllable they could hear most clearly (nonforced condition) or select the syllable they believed was presented to (b) their right ear (forced-right condition) or (c) their left ear (forced-left condition). The nonforced condition has been argued to assess language lateralization, whereas the two forced-attention conditions have been argued to assess cognitive and attentional control (Hugdahl et al., 2013). There were 36 trials per condition consisting of every combination of six syllables used as verbal stimuli (“ba,” “ta,” “ka,” “da,” “ga,” “pa”; each lasted ~350 ms); homonymous trials (in which the same syllable was presented to each ear) were not used for analysis other than as a data-quality check. Participants responded with a button press after each trial. The nonforced condition was always presented first, and the order of the forced-left and forced-right conditions was counterbalanced across participants. The primary outcome variable for this task was the number of identified syllables that were presented to each ear in each condition. As in previous research (Hugdahl et al., 2013), for both studies, we analyzed these task data using a 3 (task condition) × 2 (ear) × 2 (group) analysis of variance (ANOVA), expecting that a significant interaction would indicate that differential allocation of attention to different ears may be impaired in the voice-hearing groups.

The inhibition of currently irrelevant memories (ICIM) task consists of three blocks: a continuous recognition block and two inhibition blocks. In the first block, participants were presented with a series of black-and-white line drawings. They were asked whether each item had been previously presented and responded with a button press. In the second and third blocks, participants were instructed to forget the images they had seen so far. They were then asked whether each item had been previously presented within the second/third block only, and they responded with a button press. The second and third blocks therefore required intentional inhibition of items presented in earlier blocks. Images were displayed in the center of the computer screen for 2,000 ms (interstimulus interval = 700 ms), and participants were required to respond with a button press within this time. There was a timed 30-s break between Blocks 1 and 2 and a 5-min break between Blocks 2 and 3, during which time participants completed questionnaires. Each block contained the same images (60 unique images in total). Within each block, there were 95 trials: 40 images were presented once, five were presented twice, and 15 were presented three times. There were therefore 60 opportunities to make a false alarm response (i.e., respond that an image had been repeated when it had not). The primary outcome variable from this task was the number of false alarm responses made in each block. As an alternative measure of performance, temporal-context confusion (TCC) was calculated. TCC measures the extent to which participants confuse information between task blocks taking into account Block 1 performance (continuous recognition performance). It is calculated as follows:

$$\frac{Run\;2\;FAs}{Run\;2\;Hits}-\frac{Run\;1\;FAs}{Run\;1\;Hits},$$

where FA = false alarm.

Study 1 used only the first two blocks of the task to shorten the testing session and lessen fatigue in the patient group. Study 2 used all three blocks of the task. Data were analyzed using a 2 (task block) × 2 (group) ANOVA for Study 1 and a 3 (task block) × 2 (group) ANOVA for Study 2; the number of false alarms was the dependent variable. We expected to observe a larger increase in false alarms in the second block in the voice-hearing groups compared with control participants.

For all tasks, in the event of nonnormally distributed data, log-transformation was attempted. In all cases, this did not improve normality; therefore, nonparametric tests were used when possible. If no significant difference between groups was evident, Bayesian *t* tests using default Cauchy priors are reported to assess the strength of evidence for the null hypothesis.

### Procedure

Testing took place in a quiet room either at the participant’s home, in a health care setting, or in a university room. Sessions lasted 45 to 60 min. The voice-hearing groups in both studies were also interviewed regarding voice phenomenology (these results are reported elsewhere; Alderson-Day et al., 2021), typically around 1 week before testing, at which PSYRATS data were gathered. Procedures were approved by a university ethics committee and local health research authorities (Study 1).

## Results

### Study 1: patients compared with control participants

#### Assessment of hallucinations

The patient group scored higher than control participants on all measures of hallucination proneness and delusional ideation (Table 2). PSYRATS subscale scores can be found in Table 2.

**Table 2.** Assessments of Hallucinations and Delusional Ideation in Studies 1 and 2

Variable	Study 1				Study 2			
	Patients	Control participants	*M* [95% CI]	Cohen’s *d*	NCVHs	Non-voice-hearer control participants	*M* [95% CI]	Cohen’s *d*
PSYRATS								
Physical (0–16)	9.42 (2.29)	—	—	—	5.31 (2.87)	—	—	—
Cognitive (0–12)	6.52 (1.59)	—	—	—	3.92 (2.28)	—	—	—
Emotional (0–16)	9.94 (3.92)	—	—	—	0.08 (0.39)	—	—	—
LSHS								
Auditory (5–20)	11.75 (3.17)	7.64 (2.13)	**4.11** **[2.73, 5.50]**	1.56	10.60 (2.97)	7.16 (1.84)	**3.44** **[2.04, 4.83]**	1.38
Visual (4–16)	8.37 (3.43)	5.18 (1.74)	**3.19** **[1.81, 4.57]**	1.22	6.27 (2.03)	4.96 (1.51)	**1.31** **[0.30, 2.32]**	0.73
PDI								
Sum (0–21)	10.79 (5.92)	6.06 (4.47)	**4.73** **[2.02, 7.43]**	0.92	5.20 (2.92)	3.96 (2.88)	1.24 [−0.41, 2.89]	0.43
Distress (0–84)	39.53 (25.42)	14.48 (11.54)	**25.05** **[15.11, 34.99]**	1.32	7.54 (7.60)	8.04 (7.37)	0.51 [−3.76, 4.76]	−0.07
Conviction (0–84)	39.36 (27.86)	17.11 (12.86)	**22.25** **[11.33, 33.18]**	1.07	17.49 (14.71)	11.32 (9.08)	6.17 [−0.78, 13.12]	0.51
Preoccupation (0–84)	37.30 (25.62)	14.19 (11.02)	**23.11** **[13.20, 33.02]**	1.23	9.21 (9.33)	8.12 (7.99)	1.09 [−3.85, 6.03]	0.13
HADS								
Anxiety (0–42)	—	—	—	—	4.19 (2.83)	4.92 (3.74)	0.73 [−1.12, 2.58]	−0.22
Depression (0–42)	—	—	—	—	1.42 (1.42)	2.19 (2.33)	0.77 [−0.31, 1.85]	−0.40

Note: The values after each scale name represent the range of possible scores. Means represent mean difference between groups with standard deviation in parentheses. The 95% confidence intervals (CIs) represent the interval around the mean difference between groups. Boldface type indicates 95% CIs that do not cross 0. NCVHs = nonclinical voice hearers; PSYRATS = Psychotic Symptom Rating Scale (Haddock et al., 1999); LSHS = Launay-Slade Hallucination Scale (nine items; Bentall & Slade, 1985; McCarthy-Jones & Fernyhough, 2011); PDI = Peters Delusion Inventory (21 items; Peters et al., 2004); HADS = Hospital Anxiety and Depression Scale (Zigmond & Snaith, 1983).

#### Auditory SD

**Fig. 1.**
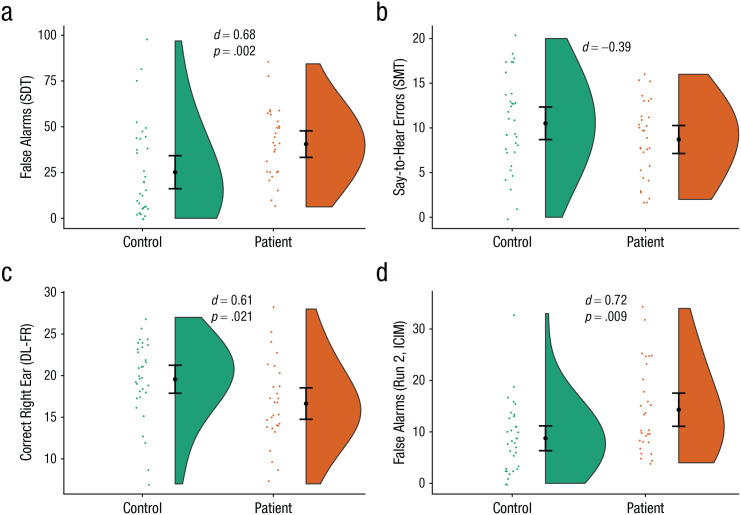
Task performance in patient group and control participants in Study 1. The graph in (a) shows the number of false alarms in the auditory signal detection task (SDT). The graph in (b) shows the number of say-to-hear errors in the source-memory task (SMT). The graph in (c) shows the number of correct right-ear responses in the forced-right (FR) condition of the dichotic-listening (DL) task. The graph in (d) shows the number of false alarms in the second block of the task involving inhibition of currently irrelevant memories (ICIM). Negative effect sizes represent results in the direction opposite the hypothesized results. Black heavy dots represent means, and error bars represent 95% confidence intervals. Colored dots represent individual data points. The shaded areas represent the probability distributions.

As predicted, the patient group had a significantly higher false alarm rate (*M* = 40.52, *SD* = 19.04) on the SD task than control participants (*M* = 25.19, *SD* = 25.43; *U* = 256.5, *p* = .002, *d* = 0.68; see Fig. 1a). Secondary analysis using SD parameters showed that the patient group (*M* = 0.96, *SD* = 0.59) had a lower response β than control participants (*M* = 2.64, *SD* = 2.84; *U* = 287.5, *p* = .007, *d* = 0.80)—indicating a greater tendency to say speech was present—and lower sensitivity (*d*′), indicating reduced accuracy at detecting speech (patient group: *M* = 1.02, *SD* = 0.43; control participants: *M* = 1.49, *SD* = 0.61; *U* = 247, *p* = .001, *d* = 0.89).

#### DL

There was a main effect of ear, *F*(1, 58) = 30.76, *p* < .001, η_*p*_^2^ = .347, which indicates a right ear advantage across both samples. There was also a main effect of condition, *F*(2, 116) = 7.85, *p* < .001, η_*p*_^2^ = .119, although not a significant main effect of group, *F*(1, 58) = 3.98, *p* = .051, η_*p*_^2^ = .064. There was no interaction between condition and group, *F*(2, 116) = 0.63, *p* = .536, η_*p*_^2^ = .011, but there was an interaction between ear and condition, *F*(2, 116) = 112.16, *p* < .001, η_*p*_^2^ = .659, which indicates orienting of attention according to the instructions in each condition across all participants.

There was a three-way interaction between condition, ear, and group, *F*(2, 116) = 3.91, *p* = .023, η_*p*_^2^ = .06. To explore this interaction further, we conducted three 2 × 2 ANOVAs (for the three conditions). In the nonforced and forced-left conditions, there was no interaction between ear and group; nonforced: *F*(1, 58) = 0.35, *p* = .56, η_*p*_^2^ = .01; forced-left: *F*(1, 58) = 1.93, *p* = .170, η_*p*_^2^ = .03. In the forced-right condition, there was a significant interaction between ear and group, *F*(1, 58) = 5.26, *p* = .025, η_*p*_^2^ = .08, although, notably, this test would not have been significant with a corrected α level (.05/3 = .017). Finally, we conducted two independent samples *t* tests (corrected α level = .05/2 = .025) using data from the forced-right condition, which indicated that control participants (*M* = 19.56, *SD* = 4.66) made more correct responses in the right ear than the patient group (*M* = 16.64, *SD* = 4.88), *t*(58) = 2.37, *p* = .021, *d* = 0.61, whereas the patient group made more correct left ear responses (*M* = 7.21, *SD* = 2.96) than control participants (*M* = 5.84, *SD* = 2.95), although not at a statistically significant level, *t*(58) = 1.79, *p* = .078, *d* = 0.46.

#### Intentional inhibition

Across all participants, there was a significant effect of task block; there were more false alarms in the second block (*M* = 11.35, *SD* = 8.07) than in the first block (*M* = 4.81, *SD* = 5.14), *F*(1, 60) = 57.02, *p* < .001, η_*p*_^2^ = .49, which indicates failures of intentional inhibition in the latter stage of the task, as expected. There was a significant effect of group; the patient group (*M* = 20.17, *SD* = 11.93) made more false alarms than the control group (*M* = 12.64, *SD* = 10.04) across the whole task, *F*(1, 60) = 7.30, *p* = .009, η_*p*_^2^ = .11. There was also a significant interaction between task block and group, *F*(1, 60) = 4.09, *p* = .048, η_*p*_^2^ = .06. Two Mann-Whitney *U* tests (corrected α = .05/2 = .025) showed a significant difference between groups in the second task block (*U* = 294.5, *p* = .009, *d* = 0.73) but not the first block (*U* = 350.5, *p* = .07, *d* = 0.39). Using the TCC measure, we found that the patient group showed a significantly higher score (*M* = 0.30, *SD* = 0.41) than control participants (*M* = 0.17, *SD* = 0.20; *U* = 316, *p* = .02, *d* = 0.41), which indicates more confusion between task blocks.

#### RM

An ANCOVA with say-to-hear errors as the dependent variable and new-to-hear errors as a covariate (to adjust for guessing; Woodward et al., 2007) indicated no significant effect of group, *F*(1, 60) = 3.47, *p* = .067, η_*p*_^2^ = .05. Patients made numerically fewer say-to-hear errors (*M* = 8.70, *SD* = 4.20) than control participants (*M* = 10.52, *SD* = 5.14), in contrast to our hypothesis. This difference was also not significant without inclusion of the covariate, *t*(61) = 1.53, *p* = .132, *d* = 0.38; Bayesian *t* tests (Bayes’s factor [BF]) indicated evidence in favor of the null (BF_10_ = 0.11). Likewise, further analysis with overall source accuracy (proportion of words that were correctly recalled as old for which the source was also correctly recalled), *t*(61) = 0.48, *p* = .636, *d* = 0.12, and old-new accuracy, *t*(61) = 1.49, *p* = .142, *d* = 0.38, showed no significant difference between groups.

### Study 2: NCVHs compared with non-voice-hearing control participants

#### Assessment of hallucinations

The NCVH group scored higher than control participants on self-report assessments of hallucinations (LSHS). There were very large differences in reports of auditory hallucinations and a lesser difference in visual hallucinations (see Table 2). Note that only one participant in the NCVH group reported any distress linked to the voices (assessed using the PSYRATS). Differences between the groups in delusional ideation (PDI) were small, and all confidence intervals crossed 0. Likewise, differences between the groups in levels of anxiety and depression (HADS) were small, and confidence intervals crossed 0 (the NCVH group scored slightly lower than control participants; see Table 2).

#### Auditory SD

**Fig. 2.**
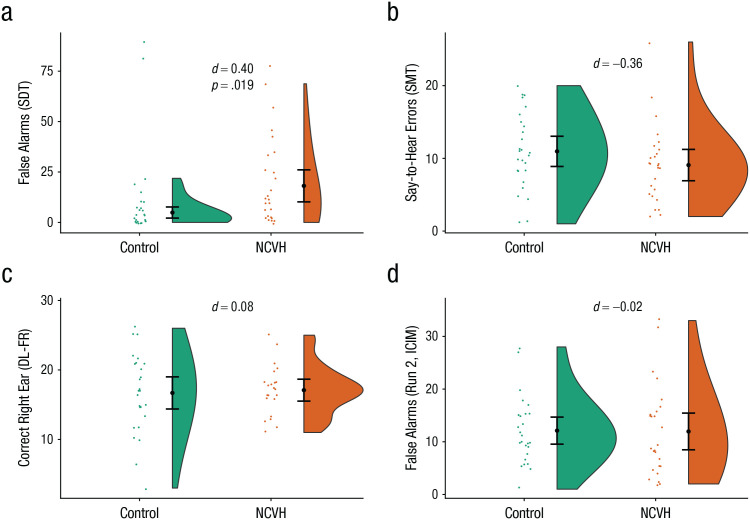
Task performance in nonclinical voice hearers (NCVHs) and non-voice-hearing control participants in Study 2. The graph in (a) shows the number of false alarms in the auditory signal detection task (SDT). The graph in (b) shows the number of say-to-hear errors in the source-memory task (SMT). The graph in (c) shows the number of correct right-ear responses in the forced-right (FR) condition of the dichotic-listening (DL) task. The graph in (d) shows the number of false alarms in the second block of the task involving inhibition of currently irrelevant memories (ICIM). Negative effect sizes represent results in the direction opposite the hypothesized results. Black heavy dots represent means, and error bars represent 95% confidence intervals. Colored dots represent individual data points. The shaded areas represent the probability distributions.

The NCVH group (*M* = 20.43, *SD* = 22.22) had a significantly higher false alarm rate than the non-voice-hearing control participants (*M* = 11.38, *SD* = 23.30; *U* = 202.0, *p* = .019, *d* = −0.40; see Fig. 2a). Further analysis using SD parameters showed that the NCVH group had a lower β; *M* = 2.79, *SD* = 2.97) than control participants (*M* = 4.99, *SD* = 3.60; *U* = 201.0, *p* = .020, *d* = 0.67) and also lower sensitivity (*d*′; NCVH group: *M* = 1.95, *SD* = 0.58; control participants: *M* = 2.27, *SD* = 0.59; *U* = 177.0, *p* = .005, *d* = 0.55).

#### DL

There was a main effect of ear, *F*(1, 46) = 29.62, *p* < .001, η_*p*_^2^ = .392, which indicates a right ear advantage across both samples. There was also a main effect of condition, *F*(2, 92) = 5.23, *p* = .007, η_*p*_^2^ = .102, although no significant main effect of group, *F*(1, 46) = 0.14, *p* = .707, η_*p*_^2^ = .003. There was not a significant interaction between condition and group, *F*(2, 92) = 2.45, *p* = .092, η_*p*_^2^ = .051, but there was an interaction between ear and condition, *F*(2, 92) = 38.78, *p* < .001, η_*p*_^2^ = .457, which indicates orienting of attention according to the instructions in each condition across all participants.

The three-way interaction was not significant, *F*(2, 92) = 1.75, *p* = .179, η_*p*_^2^ = .037, and no other main effects or interactions including group were significant (all *p*s > .092). Bayesian *t* tests indicated evidence in favor of the null for the nonforced condition (BF_10_ = 0.36) and forced-right (BF_10_ = 0.24) condition but were equivocal in distinguishing between the null and alternative hypothesis in the forced-left condition (BF_10_ = 0.99).

#### Intentional inhibition

Across both groups, there was a significant effect of task block with more false alarms in the second block (*M* = 12.04, *SD* = 7.50) and, to a lesser extent, the third block (*M* = 8.63, *SD* = 6.31) than the first block (*M* = 4.13, *SD* = 4.09), *F*(2, 100) = 44.09, *p* < .001, η_*p*_^2^ = .47, which indicates failures of intentional inhibition in the latter blocks. The pattern of fewer false alarms in the third block than the second block is consistent with previous research (Alderson-Day et al., 2019) and reflects the longer time span between Blocks 2 and 3. There was no main effect of group, *F*(1, 50) = 0.01, *p* = .942, η_*p*_^2^ < .001, and no interaction between task block and group, *F*(2, 100) = 0.49, *p* = .613, η_*p*_^2^ = .01, which indicates that the NCVH group was no more likely to make false alarms in the inhibition blocks than control participants. Bayesian *t* tests indicated support for the null hypothesis for Run 2 (BF_10_ = 0.26) and Run 3 (BF_10_ = 0.22). Using the TCC measure, we found that there was not a significant difference between the NCVH group (*M* = 0.26, *SD* = 0.24) and the control participants (*M* = 0.28, *SD* = 0.20; *U* = 298.5, *p* = .475, *d* = 0.09).

#### RM

An ANCOVA with say-to-hear errors as dependent variable, group as independent variable, and new-to-hear errors as covariate indicated no significant effect of group, *F*(1, 50) = 2.05, *p* = .158, η_*p*_^2^ = .04. There was also not a significant difference between groups when the covariate was not included, *t*(51) = 1.30, *p* = .201, *d* = 0.36, which indicates no difference between the NCVH group and the control group. Bayesian *t* tests indicated support for the null hypothesis (BF_10_ = 0.13). There was no difference between groups in overall source accuracy, *t*(51) = 0.10, *p* = .92, *d* = 0.03, or old-new recognition, *t*(51) = 1.25, *p* = .217, *d* = 0.34.

## Discussion

This study provides evidence for key similarities and differences in the cognitive profiles of voice-hearing psychosis patients and NCVHs (for a summary, see Table 3). Across the two studies, we showed that the patient group and NCVHs had a lower criterion and lower sensitivity on an auditory SD task than control participants, which reflects a higher false alarm rate and, to a lesser extent, a higher hit rate. In the patient group, we partially replicated previous results regarding impaired attentional control in a DL paradigm (Hugdahl et al., 2013) and lower performance on an intentional inhibition task (Waters et al., 2006). These patterns of performance were not found in the NCVH group, however, who did not differ from control participants. Finally, we did not replicate previous findings regarding an externalizing bias in RM (Brookwell et al., 2013); neither voice-hearing group differed from their respective control groups. Our findings therefore suggest that biases in auditory SD seem to be associated with hallucinations specifically (rather than psychopathology more broadly), whereas impaired intentional inhibition and attentional control might be associated with psychosis more broadly—and potentially play a role in attributes of hallucinations that cause them to be distressing or clinically relevant. Our study is the first, to our knowledge, to use tasks across a number of domains (RM, intentional inhibition, SD, DL) within the same studies and to use these tasks within an NCVH group. These findings raise important issues regarding (a) the underlying cognitive mechanisms of AVHs and (b) continuity and discontinuity between clinically relevant and nonclinical hallucinations.

**Table 3.** Summary of Findings in Studies 1 and 2

Study	Signal detection	Dichotic listening	Intentional inhibition	Reality monitoring
Study 1 (patient group)	**0.68**	**0.61**	**0.72**	−0.39
Study 2 (NCVH group)	**0.40**	0.08	−0.02	−0.36

Note: Numbers indicate effect sizes (*d*) for the comparison between the voice-hearing group and the control group in each study for each task. Negative effect sizes indicate opposite directionality to hypothesized. Boldface type indicates statistically significant comparisons. NCVH = nonclinical voice hearer.

Biased SD performance, observed in both of the present studies, may underlie AVHs across clinical and nonclinical populations. This is consistent with a recent large general-population study showing that biased SD task performance was associated with the number of hallucinatory experiences reported in the general population (Chinchani et al., 2021; Moseley et al., 2021). Together with meta-analytic evidence (Brookwell et al., 2013), there is strong evidence that SD biases are associated with hallucinations regardless of clinical status and may track across the psychosis continuum. Theoretically, this is consistent with arguments regarding overweighted top-down processes and the role of strong speech priors (e.g., Corlett et al., 2019) and with neuroimaging studies showing activation in brain areas associated with auditory perception (Jardri et al., 2011), although more work is required to understand which aspects of this task drive the association (e.g., verbal imagery; Moseley et al., 2016). Our findings also indicated lower sensitivity in both voice-hearing groups. One possible explanation for this regards the association between hearing impairment and hallucinations (Linszen et al., 2019), although this has not been systematically explored in relation to the SD task in hallucinations research. Further research into bottom-up processes (e.g., with audiometric testing) alongside cognitive tasks could test any mediating role.

A key insight provided from the two studies reported here concerns the role of memory inhibition. Our data indicate that lower performance on the ICIM task may be specific to psychotic hallucinations rather than vary across a continuum. This is in contrast to previous studies indicating that ICIM performance was associated with hallucination proneness in the general population (Alderson-Day et al., 2019; Paulik et al., 2007) and with theorizing regarding continuity between clinical and nonclinical groups in inhibitory ability (Badcock & Hugdahl, 2012). Likewise, our data indicated atypical attentional control on the DL task in the patient group (reflected in differences in performance in the forced-right condition but not the nonforced condition) but not the NCVH group. Intact inhibitory ability and attentional control in NCVHs may be reflected in higher level of control over voices compared with individuals with psychosis. A fruitful area for future research would be to examine associations between specific attributes of hallucinations—for example, volitional control, which differs across clinical and nonclinical groups (Swyer & Powers, 2020)—and specific cognitive domains, such as intentional inhibition of memories. Given our findings, it could be hypothesized that performance on the ICIM task may be associated with reported control over voices. Alternatively, intact intentional inhibition ability observed here could reflect other clinically relevant potential differences between the groups, for example, childhood trauma (Bailey et al., 2018). Future research should investigate whether factors such as trauma could mediate the association between intentional inhibition and hallucinations or psychosis more generally.

Finally, we observed no difference in RM performance (using the source-memory task) between either of the voice-hearing groups and control participants. RM has arguably been the domain most frequently associated with hallucinations in cognitive models (Brookwell et al., 2013; Waters et al., 2012), but the evidence regarding associations between task performance and hallucinations is mixed given that some studies show associations in psychosis (Woodward et al., 2007) and associations with hallucination proneness in the general population (Laroi et al., 2004) but more recent studies have failed to replicate this finding using multiple different variants of the source-memory task (Alderson-Day et al., 2019; Garrison et al., 2017). In particular, a large multisite general-population study failed to find an association between hallucinatory experiences and RM (Moseley et al., 2021). There is therefore increasing evidence that this may not be a key cognitive mechanism associated with AVHs. An unexplored alternative is that source-monitoring biases may be evident only in psychosis patients with longer-term histories of illness and wider difficulties with functioning.

Our findings are of particular relevance to discussions of the continuum hypothesis as applied to hallucinations. One (simplistic) model of the continuum, assuming continuity of cognitive processes, could be that psychosis patients sit at the extreme end of a continuum, NCVHs are lower down the continuum, and individuals in the general population who report occasional hallucinatory experiences are lower still (Baumeister et al., 2017). To some extent, our data with the SD task may support this given that the patient group showed a difference from control participants with a large effect size and the NCVH group a difference with a medium effect size—and a recent general-population study (Moseley et al., 2021) showed a small effect size). However, as noted, memory inhibition and attentional control did not appear to vary continuously in this fashion, which suggests discontinuity between AVHs in psychosis and nonclinical variants. As others have suggested, this complexity could point to multiple continua (Waters & Fernyhough, 2019) with variations in, for example, distress, control, associated dysfunction (e.g., delusional frameworks), and neurodevelopmental structural brain changes (Garrison et al., 2019; Powers et al., 2020). An alternative viewpoint might be that although clinical and nonclinical AVHs share some core cognitive components, they differ in terms of the kinds of cognitive mechanism drawn on rather than varying continuously at a cognitive level. Viewed from this perspective, clinical and nonclinical hallucinations are not fundamentally different in kind, but a continuum might not be the best model at the level of cognition. Providing an answer to this question will require larger-scale studies of cognition in both clinical and nonclinical samples.

There are a number of limitations to the two studies reported here. First, both groups represent only one of many potential samples of clinical voice hearers and NCVHs—that is, the patient group members were early intervention service users, and NCVHs all reported spiritual interpretations of their voices. Further studies should seek to recruit and compare a variety of voice hearers—for example, NCVHs without spiritual interpretations may differ in important ways (e.g., control over voices, cultural and social background) to the sample reported here. As noted previously, much research into NCVHs has focused on similar groups (i.e., individuals with spiritual or paranormal interpretations of their voices; Peters et al., 2016; Powers, Kelley, & Corlett, 2017). The preponderance of spiritual beliefs in NCVH participants in the research literature could reflect a key element of their “nonclinical” status—that is, such beliefs may play a protective role, helping individuals exert control or influence over voices. Second, the NCVH group scored somewhat lower on a standardized assessment of AVHs (PSYRATS) and delusions (PDI) than the patient group, which could feasibly account for differences in cognitive variables across the two studies. That said, one strength of the findings is that the NCVH group did not score notably higher than non-voice-hearing control participants on other assessments of psychopathology that would typically be heightened in psychosis (delusional ideation, anxiety, depression), which indicates that group differences in SD task performance were unlikely to be reflective of other psychopathological variables. The voice-hearing groups also scored lower than control participants on MR and the NART (assessing nonverbal and verbal intelligence, respectively), as indicated by nonoverlapping confidence intervals. It could therefore be argued that the voice-hearing groups showed a general cognitive deficit rather than deficits in any specific domains. However, given that both voice-hearing groups showed lower MR/NART performance yet showed divergent performance on other cognitive tasks, a general cognitive deficit does not seem to be the simplest explanation for the observed pattern of results. Likewise, the observation that neither group showed lower scores on the source-memory task (assessing RM) indicates some level of specificity.

Third, the sample sizes in both studies were powered to detect large effect sizes on the basis of findings from previous research (Brookwell et al., 2013), which means that the study would have been underpowered to detect smaller effects; future research could use a multisite approach (Moseley et al., 2021) to collect larger samples in these hard-to-recruit populations. This might be particularly important when recruiting NCVHs, who may show more subtle biases or impairments associated with less frequent and distressing experiences. In particular, future research with larger sample sizes could aim to recruit psychosis patients and NCVHs matched on relevant demographic attributes (e.g., age) into the same study. That said, it is possible that this approach would lead to nonrepresentative samples—that is, it might be that NCVHs are, on average, older than patients with psychosis (Peters et al., 2016; Powers, Kelley, & Corlett, 2017), and this could be a key attribute of the group. Artificially selecting for age could mask other important group differences. Future research with larger samples would allow variables such as age to be investigated in relation to variation in cognition across groups.

Fourth, the two studies were designed and conducted separately and by necessity were conducted with slightly different measures (e.g., a separately calibrated SD task because of variability in age across the groups), which limits some of our conclusions. Nevertheless, we believe the core measures are sufficiently comparable to provide meaningful inferences regarding differences between groups on key cognitive mechanisms for the first time. Furthermore, the voice-hearing groups differed on a number of demographics, notably age (NCVHs mainly reflecting older adults, as in previous studies; Powers, Kelley, & Corlett, 2017); that said, the control participants were well matched on these demographics in the two studies. Further research is needed to explore trajectories of voices and their associated cognitive processes over time. Finally, the tasks we used represent only one variant of a number that have been used in the psychosis literature, and it is possible that different variants would give different findings (e.g., many source-monitoring articles have increased cognitive load associated with self-generation; Woodward et al., 2007). A greater understanding of cognitive processes such as those presented here will undoubtedly feed into lower-level mechanistic explanations of hallucinations (e.g., the predictive processing framework) and attempts to improve treatment options for people distressed by voices.

## Supplemental Material

sj-pdf-1-cpx-10.1177_21677026211059802 – Supplemental material for Continuities and Discontinuities in the Cognitive Mechanisms Associated With Clinical and Nonclinical Auditory Verbal Hallucinations Click here for additional data file. Supplemental material, sj-pdf-1-cpx-10.1177_21677026211059802 for Continuities and Discontinuities in the Cognitive Mechanisms Associated With Clinical and Nonclinical Auditory Verbal Hallucinations by Peter Moseley, Ben Alderson-Day, Stephanie Common, Guy Dodgson, Rebecca Lee, Kaja Mitrenga, Jamie Moffatt and Charles Fernyhough in Clinical Psychological Science

## Transparency

*Action Editor:* Vina Goghari

*Editor:* Jennifer L. Tackett

*Author Contributions*

P. Moseley, B. Alderson-Day, G. Dodgson, and C. Fernyhough developed the study concept and contributed to the study design. Participant recruitment and data collection was carried out by P. Moseley, B. Alderson-Day, G. Dodgson, and S. Common. Data analysis and interpretation was carried out by P. Moseley, with contributions to interpretation from all other authors. P. Moseley drafted the manuscript, and all of the authors provided revisions. All of the authors approved the final manuscript for submission.
