# Supplementary material for: Continuities and Discontinuities in the Cognitive Mechanisms Associated With Clinical and Nonclinical Auditory Verbal Hallucinations
Source: Clin Psychol Sci. 2022 Jan 17;10(4):752–66. doi: 10.1177/21677026211059802 (PMC9280701; doi:10.1177/21677026211059802)
Supplement: sj-pdf-1-cpx-10.1177_21677026211059802 – Supplemental material for Continuities and Discontinuities in the Cognitive Mechanisms Associated With Clinical and Nonclinical Auditory Verbal Hallucinations [file sj-pdf-1-cpx-10.1177_21677026211059802.pdf]

Supplemental Material

Table S1 – Patient group current diagnoses (*N* = 31)

Diagnosis	*%*
None	48.4
Unspecified psychosis	29.0
PTSD	6.5
EUPD	6.5
Depression with psychotic features	3.2
Schizophrenia	3.2
Substance-induced psychosis	3.2

Note that many patients had not yet received a diagnosis, having recently entered clinical services. In the UK, distressing symptom presence rather than fulfillment of diagnostic criteria is an entry requirement for early intervention services, and clinicians tend to hold off on making a diagnosis.

Table S2 – Patient group current medication usage (*N* = 31)
Nb. % does not sum to 100 as some participants on more than one medication

Medication	*%*
Quetiapine	22.6
Risperidone	19.4
Aripiprazole	16.1
None	12.9
Fluoxetine	12.9
Setraline	12.9
Olanzapine	9.7
Citalopram	6.5
Haloperidol	3.2
Mirtazepine	3.2

Methodological differences between studies 1 & 2

- The auditory signal detection task was calibrated separately for the two studies. That is, for each study, 10 participants (who did not participate in the main studies) were

recruited within a similar age range to that expected for the main studies, and completed a short calibration task. For each study, speech volume was then set at a level where they were detected at 25%, 50%, 75% and 95% levels, based on the calibration data. Performance varies between the two studies (e.g., with higher d' in Study 2) because the ages of participants between calibration and the main studies varied – hence, statistical comparisons are restricted to contrasts between voice-hearing and control groups throughout the paper.

- The intentional inhibition of currently irrelevant memory task (ICIM) contained two blocks for the patient group (one continuous recognition block + one inhibition block) and three blocks for the NCVH group (one continuous recognition block + two inhibition blocks). This decision was made to minimise the length of the session for the patient group, to lessen fatigue and potential drop-out (e.g., due to boredom).
- The patient group completed both the hallucinations and delusions subscales of the PSYRATS, whereas the NCVH group completed only the hallucinations subscale. It was felt both inappropriate to pathologise spiritual beliefs, and also complex to unpick what could be classed as delusional ideation, in the non-clinical group.

Participant exclusions

Participants were excluded from individual tasks if performance was below chance levels (indicating that they may have misunderstood the task or responded incorrectly). Exclusion criteria were the same as our previous preregistered study for the signal detection, dichotic listening, and source memory tasks, which can be found here: https://osf.io/cyu6j. For the ICIM, participants were excluded if they had a d' score of less than or equal to 0 (indicating at or below chance performance) in run 1. A small amount of data was also excluded due to technical difficulties resulting in lost data, or if a participant stopped participation in that task.

Study 1

Signal detection – one exclusion for a $d' < 0$ (patient group). One other exclusion due to task non-completion (patient group).

Intentional inhibition – one exclusion for a $d' < 0$ in run 1 (patient group), one other exclusion due to task non-completion (patient group).

Source memory – one exclusion due to technical difficulties resulting in missing data (patient group).

Dichotic listening – one exclusion for 100% laterality index (patient group) and one exclusion for < 50% performance on homonymous trials (patient group). Two other exclusions due to technical difficulties resulting in missing data (one patient group, one control group).

Study 2

One participant was excluded at the screening stage due to reporting high levels of current distress due to hearing a malevolent spiritual voice (and was not included in reported *n* in the manuscript).

Intentional inhibition – one exclusion for $d' < 0$ in run 1 (control group). One further exclusion due to task non-completion (control group).

Signal detection – one exclusion for $d' < 0$ (control group). Two other exclusions due to technical difficulties resulting in missing data (control group).

Dichotic listening – three exclusions for < 50% performance on homonymous trials (NCVH group). Three further exclusions (one patient, two controls) due to task non-completion or technical difficulties resulting in missing data.

Source memory – one exclusion based on task non-completion (control group).

Table S3 – associations between assessments of hallucinations and task measures (*r* [95% CI]) (Study 1, *N* = 31)

	PSYRATS (AH)	LSHS (auditory)	SDT false alarms	SMT say-hear	DL nonforced	DL forced left	DL forced right	ICIM false alarms
**PSYRATS (AH)**	—							
**LSHS (auditory)**	.62 [.31, .81]	—						
**SDT false alarms**	-.36 [.01, -.64]	-.22 [-.57, .20]	—					
**SMT say-hear**	-.15 [-.48, .22]	.010 [-.39, .40]	.26 [-.13, .58]	—				
**DL nonforced**	.11 [-.27, .46]	-.08 [-.48, .34]	.26 [-.14, .59]	.02 [-.36, .39	—			
**DL forced left**	.27 [-.11, .59]	.23 [-.20, .59]	-.17 [-.53, .23]	-.05 [-.41, .33]	.07 [-.31, .43]	—		
**DL forced right**	0.15 [-.23, .49]	-.32 [-.65, .11]	.22 [-.19, .56]	.02 [-.36, 39]	.68 [.41, .84]	.39 [.02, .67]	—	
**ICIM false alarms**	-.10 [-.46, .27]	-.03 [-.43, .38]	-.29 [-.61, .10]	-.05 [-.41, 32]	-.30 [-.61, .09]	-.29 [-.60, .10]	-.40 [-.68, .03]	—

This table presents correlations (Pearson's r) between two hallucinations measures and the cognitive task measures within the patient group only (Study 1). These are exploratory analyses, and are presented for descriptive purposes (i.e., they are not hypotheses-driven).

PSYRATS (AH) = Psychotic Symptoms Rating Scale; LSHS (auditory) = Launay-Slade Hallucination Scale (auditory subscale); SDT false alarms = signal detection task, false alarm rate; SMT say-hear = source memory task, say-to-hear errors; DL nonforced = dichotic listening, right ear responses in the nonforced condition; DL forced left = dichotic listening, left ear responses in the forced left condition; DL forced right = dichotic listening, number of right ear responses in the forced right condition; ICIM false alarms = intentional inhibition of currently irrelevant memories task, number of false alarms in run 2. Note that N may differ slightly for different variables due to exclusions (see S4, above).

Table S4 – associations between assessments of hallucinations and task measures (*r* [95% CI]) (Study 2, *N* = 26)

	PSYRATS (AH)	LSHS (auditory)	SDT false alarms	SMT say-hear	DL nonforced	DL forced left	DL forced right	ICIM false alarms
**PSYRATS (AH)**	—							
**LSHS (auditory)**	.09 [-.31, .46]	—						
**SDT false alarms**	.02 [-.37, .41]	-.04 [-.42, .35]	—					
**SMT say-hear**	.08 [-.32, .45]	.34 [-.06, .64]	-.17 [-.52, .23]	—				
**DL nonforced**	-.06 [-.47, .55]	.12 [-.32, .51]	.04 [-.39, .46]	-.25 [-.61, .19]	—			
**DL forced left**	.16 [-.28, .55]	-.11 [-.51, .33]	-.27 [-.62, .17]	.11 [-.33, .51]	-.08 [-.48, .36]	—		
**DL forced right**	.20 [-.24, .58]	-.11 [-.51, .33]	-.10 [-.50, .34]	-.19 [-.57, .25]	.65 [.31, .84]	.24 [-.21, .60]	—	
**ICIM false alarms**	-.24 [.16, -.58]	.27 [-.13, .59]	-.19 [-.54, .22]	-.06 [-.34, .44]	-.11 [-.51, .33]	-.48 [-.75, -.07]	-.42 [-.72, -.003]	—

This table presents correlations (Pearson's r) between two hallucinations measures and the cognitive task measures within the non-clinical voice-hearer group only (Study 2). These are exploratory analyses, and are presented for descriptive purposes (i.e., they are not hypotheses-driven).

PSYRATS (AH) = Psychotic Symptoms Rating Scale; LSHS (auditory) = Launay-Slade Hallucination Scale (auditory subscale); SDT false alarms = signal detection task, false alarm rate; SMT say-hear = source memory task, say-to-hear errors; DL nonforced = dichotic listening, right ear responses in the nonforced condition; DL forced left = dichotic listening, left ear responses in the forced left condition; DL forced right = dichotic listening, number of right ear responses in the forced right condition; ICIM false alarms = intentional inhibition of currently irrelevant memories task, number of false alarms in run 2. Note that N may differ slightly for different variables due to exclusions (see S4, above).
